# Structural Development Studies of Subtype-Selective Ligands for Peroxisome Proliferator-Activated Receptors (PPARs) Based on the 3,4-Disubstituted Phenylpropanoic Acid Scaffold as a Versatile Template

**DOI:** 10.1155/2008/689859

**Published:** 2008-06-15

**Authors:** Hiroyuki Miyachi, Yuichi Hashimoto

**Affiliations:** Institute of Molecular and Cellular Biosciences, University of Tokyo, Yayoi Bunkyo-ku, Tokyo 113-0032, Japan

## Abstract

Improvements in our understanding of the functions of the nuclear receptor peroxisome proliferator-activated receptor (PPAR) subtypes as pleiotropic regulators of biological responses, including lipid, lipoprotein, glucose homeostasis, inflammation, differentiation and proliferation of various cancer cells, and memory, have provided an opportunity to develop novel PPAR ligands with characteristic subtype selectivity. Such ligands are not only chemical tools to investigate the functions of PPARs, but also candidates for the treatment of PPAR-mediated diseases, including metabolic syndrome, inflammation, dementia, and cancer. This minireview summarizes our work on the design, synthesis, and pharmacological evaluation of subtype-selective PPAR agonists based on the use of 3,4-disubstituted phenylpropanoic acid as a versatile template.

## 1. NUCLEAR RECEPTORS

The nuclear receptors (NRs) form a superfamily of ligand-dependent transcription factors that control diverse aspects of reproduction, development, homeostasis, immune function, and so on. This superfamily includes the known receptors for steroid hormones, thyroid hormones, retinoid receptors and vitamin D receptor, as well as a large number of orphan receptors. The structures of NRs are composed of several functionally important regions (designated A to F). The N-terminal A/B region contains a transcriptional activation function-1 (AF-1) motif that works independently of ligand binding. The central DNA-binding region (C region) is highly conserved among the NRs and contains two zinc-finger motifs that make contact with specific nucleotide sequences, termed hormone response elements. The C-terminal part, which consists of the D, E, and F regions, is required for ligand binding and receptor dimerization. In most receptors, this region also contains a second highly conserved transcriptional activation function-2 (AF-2) motif, which is important for ligand-dependent transcription.

Based on the elucidated human genome sequence, 48 NRs are speculated to exist in humans [1]. However, the ligands have been identified for only 20 to 25 of them. The others are so-called orphan receptors, whose endogenous ligands are not known [2, 3]. Among the NRs, much attention has been focused on the peroxisome proliferator-activated receptors (PPARs) over the past two decades.

## 2. PEROXISOME PROLIFERATOR-ACTIVATED RECEPTORS

PPARs are activated by endogenous saturated and unsaturated fatty acids and their metabolites and synthetic ligands [4]. Three subtypes have been isolated to date: PPAR*α* (NR1C1), PPAR*δ* (NR1C2), and PPAR*γ* (NR1C3), and each of them appears to be differentially expressed in a tissue-specific manner. PPAR*α* is mostly expressed in tissues involved in lipid oxidation, such as liver, kidney, skeletal, cardiac muscle, and adrenal glands. PPAR*γ* is expressed in adipose tissue, macrophages, and vascular smooth muscles. In contrast to the specific distribution of PPAR*α* and PPAR*γ*, PPAR*δ* is ubiquitously expressed [5].

Upon ligand binding, PPARs heterodimerize with another nuclear receptor partner, retinoid X receptor (RXR), and the heterodimers regulate gene(s) expression by binding to specific consensus DNA sequences, called peroxisome proliferator responsive elements. These elements are a direct repeat of the hexameric AGGTCA recognition motif, separated by one nucleotide (DR1), present in the promoter region of the target genes [6].

## 3. PPARS AS REGULATORS OF METABOLIC HOMEOSTASIS

Each of the PPAR subtypes plays a pivotal role in lipid, lipoprotein, and glucose homeostasis.** PPAR*α* regulates genes involved in fatty acid uptake (such as fatty acid binding protein, FABP), *β*-oxidation (acyl-CoA oxidase), and *ω*-oxidation (cytochrome P450). It downregulates apolipoprotein C-III, a protein that inhibits triglyceride hydrolysis by lipoprotein lipase, and it also regulates genes involved in reverse cholesterol transport, such as apolipoprotein A-I and apolipoprotein A-II [7]. PPAR*γ* is a master regulator of adipocyte differentiation, but more recent molecular-biological studies have indicated that its activation is also linked to the expression of many important genes that affect energy metabolism, such as TNF-*α*, leptin, and adiponectin genes [8]. PPAR*δ* is the least well-defined subtype among the PPARs, but recent biological study has disclosed that its activation significantly increases HDL cholesterol levels, and it influences glycemic control in a primate model of metabolic syndrome [9–11]. Furthermore, its activation markedly improved glucose tolerance and insulin resistance in ob/ob mice, although the underlying mechanism remains unclear [12].

## 4. PPARS AS TEMPLATES FOR DEVELOPMENT OF VERSATILE REGULATORS

Research in the field of PPAR biology and/or pharmacology is attracting enormous interest, and the range of therapeutic potential for PPAR agonists is rapidly expanding well beyond lipid, lipoprotein, and glucose homeostasis. For example, ligand-mediated PPAR*α* activation induces expression and activation of antioxidant enzymes, such as superoxide dismutase (SOD) and glutathione peroxidase (GTP) [13]. Therefore, PPAR*α* activation blocks the synthesis and release of inflammatory cytokines, such as IL-6 and TNF-*α*. PPAR*γ* activation attenuates the expression of inducible nitric oxide (iNOS) and cyclooxygenase-2 (COX-2), as well as the production of proinflammatory cytokines [14]. Considering that PPARs are also expressed in neurons and in astrocytes, both PPAR*α* and PPAR*γ* are expected to be useful as pharmacological targets for neuroprotection in stroke and neurodegenerative diseases.

PPAR*γ* was initially noted to be highly expressed in adipose tissue, but later studies demonstrated that PPAR*γ* was also expressed widely in tumors originated from various organs. Ligand-mediated activation of PPAR*γ* inhibits cell proliferation and/or induces apoptosis or terminal differentiation, by upregulating the expression of cyclin-dependent kinase (CDK) inhibitors, including P18, P21, and P27 [15]. PPAR*γ* also promotes cell cycle arrest by inhibiting CDK activity in several tumor cell lines [16].

Angiogenesis, the formation of new blood vessels, is a critical step in solid tumor growth.** PPAR*γ* activation inhibits the expression of at least three important genes involved in the angiogenic processes, that is, VEGF, VEGF receptor 1, and urokinase plasminogen activator (uPA) [17]. Therefore, PPAR*γ* is considered as a therapeutic target for certain human malignancies.

Based on the findings that the glitazone-class antidiabetic agents and fibrate-class antidyslipidemic agents are ligands of PPAR*γ* and PPAR*α*, respectively, much research interest has been focused on these two metabolic NR subtypes as therapeutic targets for the treatment of diabetes and dyslipidemia. In contrast, although PPAR*δ* is ubiquitously distributed in a wide range of tissues and cells, research interest in PPAR*δ* has been limited. However, after 2001, the availability of PPAR$\overset{˜}{\delta }$ knockout animals and selective ligands prompted us to examine the involvement of PPAR*δ* in fatty acid metabolism, insulin resistance, reverse cholesterol transport, inflammation, and so on. Furthermore, molecular-pharmacological studies have indicated that PPAR*δ* is also involved in other biological functions, extending beyond metabolic homeostasis. PPAR*δ* is reported to play critical role(s) in wound healing. After tissue damage resulting from chemical, mechanical, and biological injury, the injured cells release proinflammatory cytokines [18, 19]. These stimulate PPAR*δ* expression, coordinating transcriptional upregulation of integrin-linked kinase and 3-phosphoinositide-dependent kinase (PDK), and repress the expression of phosphatase and tensin homolog 10 (PTEN) [20]. As a consequence, the PKB*α* activity is increased and apoptotic cascades are repressed. The resulting increased resistance to cell death helps to maintain a sufficient number of viable wound keratinocytes for re-epithelization. Therefore, PPAR*δ* is expected to be a therapeutic target for tissue injury.

The above examples remind us that PPARs are pleiotropic NRs, and the subtypes are unique, though somewhat overlapping, therapeutic targets for the treatment of not only metabolic homeostasis, but also inflammation, cancer, neurodegeneration, wounds, and so forth. PPAR ligands clearly have enormous potential as therapeutic agents, and the range of possible applications has certainly not yet been fully explored.

From a basic research point of view, it is of primary importance to develop potent and PPAR subtype-selective ligands as chemical tools to investigate individual PPAR functions in detail.** Furthermore, from a medicinal chemical point of view, it is also important to consider PPAR dual agonists (which can activate two out of three PPAR subtypes effectively), such as PPAR*α*/*δ*-, PPAR*α*/*γ*-, and PPAR*δ*/*γ* dual agonists, and PPAR-pan agonists (which can activate all three PPAR subtypes), since these may exhibit additive and/or synergistic pharmacological effects.

In this minireview, we focus on our structural development studies to create PPAR*α*-selective PPAR*α*/*δ*-dual and PPAR*δ*-selective agonists, using the 3,4-disubstituted phenylpropanoic acid template as a common structure.

## 5. OUR WORKING HYPOTHESIS CONCERNING THE LIGAND SUPERFAMILY CONCEPT

We have been engaged in structural development studies of NR ligands (agonists and antagonists) for over ten years, based on our working hypothesis concerning the NR ligand superfamily concept [21]. Broadly speaking, the structural/functional features of NRs are similar, even though there are many kinds of NRs (48 in humans), that is, NRs generally consist of an aminoterminal region which has a ligand-independent transcriptional activation function (AF-1), a DNA-binding domain (DBD) with a motif structure of two zinc fingers which has a sequence-specific DNA (response element: RE)-binding function, and a large carboxyl-terminal region (ligand-binding domain: LBD), which has a specific ligand-binding function/dimerization function/ligand-dependent activation function (AF-2) [22]. Thus, NRs are probably derived from a single ancestral protein, and may have structurally evolved in order to fit various kinds of endogenous NR ligands. We speculated that similar evolution of NR ligands would have occurred from an ancestral ligand to form a superfamily, even though NR ligands now have diverse functions. Based on this working hypothesis, we divided the structure of NR ligands into two portions. One is a common hydrophobic framework that fits into the ligand binding pocket of the basic ancestral protein, and the other is a characteristic structural motif which provides NR selectivity. If this hypothesis is correct, it is easily deduced that the hydrophobic backbones of various NR ligands should be exchangeable with each other to construct structurally new NR ligands.

**Figure 1:**
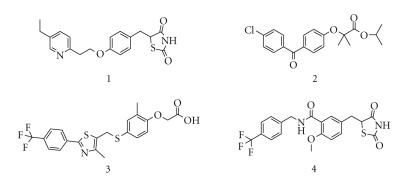
Structures of: (1) PPAR*γ* agonist pioglitazone, (2) PPAR*α* agonist fenofibrate, (3) PPAR*δ* agonist GW-501516, and (4) dual PPAR*γ*/*α* agonist KRP-297.

Numerous previous reports have indicated that PPAR subtype-selective agonists have certain unique structures associated with the subtype selectivity. For example, thiazolidine-2,4-dione, and related structures, as exemplified by pioglitazone (1), for PPAR*γ* selective agonists, 2,2-dialkyl (usually dimethyl) phenoxyacetic acid structure, exemplified by fenofibrate (2), for PPAR*α*-selective agonists, and 2,2-unsubstituted phenoxyacetic acid structure, exemplified by GW-501516 (3), for PPAR*δ*-selective agonist (Figure 1). But, based on our working hypothesis, we anticipated that various kinds of subtype-selective, dual-, and pan-agonists could be created by using a common chemical framework as a template.

## 6. PPAR*α*-SELECTIVE AGONIST: KCL

**Scheme 1:**
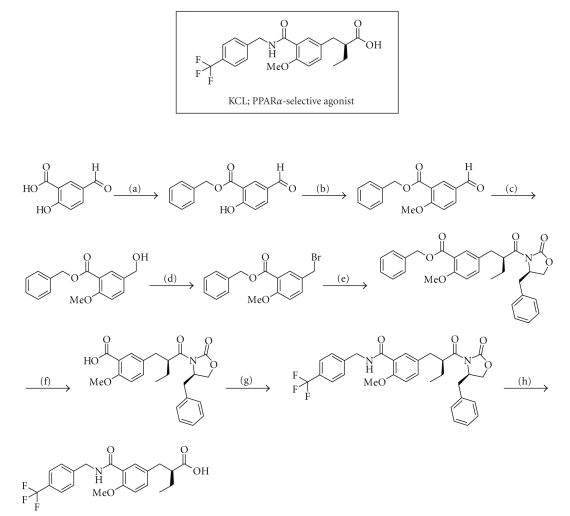
Chemical structure of our PPAR*α*-selective agonist KCL, and the synthetic route. Reagents and conditions: (a) BnBr, KHCO_3_, DMF, rt., (b) MeI, K_2_CO_3_, DMF, rt. (c) NaBH_4_, EtOH, rt. (d) PBr_3_, ether, 0°C, (e) (1) (*R*)-4-benzyl-3-butyryloxazolidin-2-one, LiHMDS, THF, −40°C−−10°C, (2) benzyl 5-bromomethyl-2-methoxybenzoate, THF, −40°C−−10°C, (f) H_2_, 10% Pd–C, AcOEt, rt., (g) (1) ethyl chloroformate, TEA, THF, −10°C, (2) 4-trifluoromethylbenzylamine, THF, −10°C -rt., (h) LiOH H_2_O, H_2_O_2_, THF–H_2_O, 0°C.

**Figure 2:**
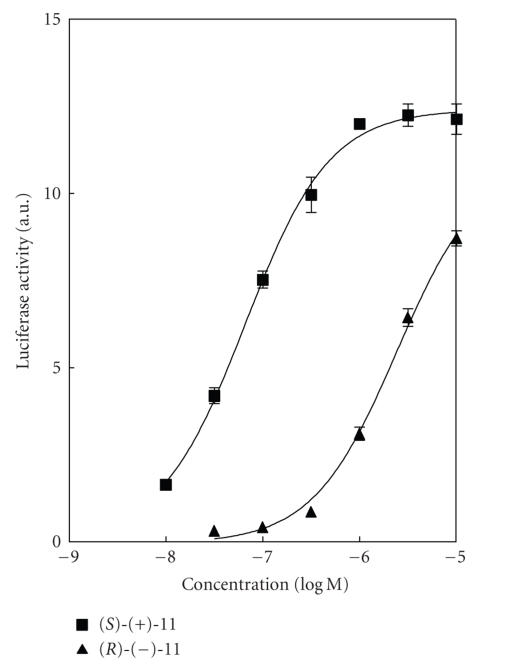
Dose-response curves of each enantiomer for PPAR*α* transactivation.

We designed and synthesized a series of substituted phenylpropanoic acid derivatives in order to develop structurally new human PPAR*α*-selective agonists, using KRP-297 (4), a unique thiazolidine-2,4-dione derivative with PPAR*γ*/*α* dual agonist activity, as a lead compound for the preparation of antidyslipidemic agents [23–25]. Although KRP-297 belongs structurally to the glitazones (thiazolidine-2,4-dione class insulin sensitizers), it binds directly to and activates not only PPAR*γ*, but also PPAR*α* with almost equal affinity (its affinity for PPAR*γ* and PPAR*α* is approximately 0.23 and 0.33 *μ*M, resp., and it transactivates PPAR*γ* and PPAR*α* with effective concentrations of 0.8 and 1.0 *μ*M, resp.) [24]. This is a characteristic feature of classical glitazones, including troglitazone (5), pioglitazone (1), and rosiglitazone (6), which were reported to bind to and activate selectively the PPAR*γ* subtype. The reason why they exhibit dual-ligand nature was unclear, but we anticipated that the replacement of the thiazolidine-2,4-dione ring structure of KRP-297 with another acidic functionality, such as a carboxyl group, which is usually used in fibrates, might favor PPAR*α* selectivity and that further chemical modification might improve the potency and selectivity for the PPAR*α* subtype. Structurally, KRP-297 can be regarded as having three key regions: (i) the acidic head group, (ii) the linking group, and (iii) the hydrophobic tail group. We performed chemical modification of the (i) to (ii) parts of the molecule to understand the structure-activity relationship in detail. After synthesizing numerous compounds, we obtained KCL, (*S*)-2-[4-methoxy-3-(4-trifluoromethylbenzylcarbamoyl)phenylmethyl]butyric acid (*S*)-11 [26–29] as a potent and PPAR*α*-selective agonist. The synthetic route to KCL is shown in Scheme 1. Our SAR results can be summarized as follows. (i) The distance between the carboxyl group and the right-side benzene ring in the compound is important. (ii) The introduction of alkyl substituents at the *α*-position of the carboxyl group strikingly affected PPAR*α* transactivation activity and subtype selectivity, and the ethyl group is the most favorable (Table 1). (iii) Stereochemistry at the *α*-position of the ethyl group is crucial and the (*S*)-configuration is preferable (Figure 2). (iv) The length of the linking group is important for potency, and a three-atom unit with an amide group such as –CH_2_–NH–CO– is preferable (Table 2).

(Although compound 29 exhibited potent and PPAR*α*-selective activity, we did not go forward with this compound, because our experience indicated that it would be metabolically unstable; consequently it was not expected to exhibit potent or prolonged in vivo activity, despite its potency in vitro.)

It is important to note that KCL exhibits distinct species-dependence in transactivation for PPAR*α*. Although species dependency of some PPAR*α* agonists was reported previously, the degree of selectivity was low [30, 31]. However, the species-selectivity of KCL is extremely high: KCL activated human, dog, and rat PPAR*α* with EC_50_ values of 0.06, 0.16, and 5.2 *μ*M, respectively (Table 3). KCL exhibited species preference for humans and its transactivation activity for PPAR*α* was approximately 100-fold and 30-fold less potent in rats than in humans and in dogs, respectively.

This apparent species difference was reported to result from specific interaction between the 272-aminoacid, isoleucine (Ile272), which is located on the helix 3 region of the human PPAR*α* LBD and the hydrophobic tail part of KCL [32–34]. The corresponding aminoacid residue in the rat PPAR*α* LBD is sterically more bulky phenylalanine. KCL was reported to reduce plasma triglyceride levels >100-fold more potently in dogs than in rats, which is consistent with the in vitro assay data. Clinical studies of a KCL-related compound are under way.

## 7. PPAR*α*/*δ*-DUAL AGONIST: TIPP-401

We next planned to develop a PPAR*α*/*δ*-dual agonist, which would effectively activate both PPAR*α* and PPAR*δ*, because pharmacological evidence indicated that PPAR*α* regulates the expression of genes encoding proteins involved in lipid and lipoprotein homeostasis, and subsequent pharmacological findings for PPAR*δ* demonstrated that it also plays a key role in lipid metabolism and insulin resistance. Furthermore, PPAR*δ* plays a key role in foam cell and macrophage activation in atherosclerosis.

**Figure 3:**
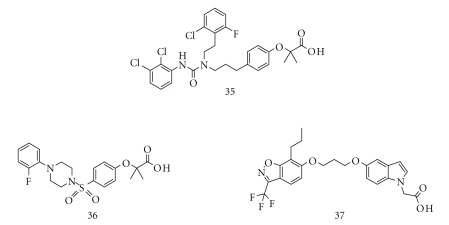
Structures of the dual PPAR*α*/*δ* agonists.

The metabolic function(s) of PPAR*δ* seem to be mainly targeted to adipose tissue and smooth muscle, via fatty acid oxidation and energy uncoupling. If this were the case, we expected that a compound which can effectively activate both PPAR*α* and PPAR*δ* might have additive and/or synergistic positive effect(s) in the treatment of metabolic syndrome, by modulating both hepatic fatty acid oxidation through PPAR*α*, and fatty acid oxidation and energy uncoupling in muscle and adipose tissue through PPAR*δ*. In addition, in 2004, there were only a few examples of PPAR*α*/*δ* dual agonists in the literature, including compounds 35 [35], 36 [36], and 37 [37] (Figure 3), and their activities seemed rather low and their structural variety poor. Therefore, there was considerable interest in creating novel PPAR*α*/*δ* dual agonists from both basic scientific and clinical points of view. We expected that small manipulations of the structure of KCL would affect the activities towards both PPAR*α* and PPAR*δ*. Therefore, we reconsidered the SAR of PPAR*α*-selective KCL derivatives.

As the linking group of the KCL series, we found that a three-atom unit with an amide group such as –CH_2_–NH–CO– was preferable for potency and selectivity against PPAR*α*, and these compounds did not exhibit remarkable PPAR*δ* activity. We noted that a flexible linker, such as –CH_2_–CH_2_–CH_2_– (31) or –CH_2_–CH_2_–O– (32), decreased PPAR*α* transactivation activity, but also resulted in the appearance of PPAR*δ* transactivation activity. Therefore, we focused our attention on a hybrid-type linker, that is, –CO–NH–CH_2_– (33), and found that this linker increased both PPAR*α* and PPAR*δ* transactivation activity to some extent, as compared with the amide-type (–CH_2_–NH–CO–) linker in KCL (Table 2).

**Scheme 2:**
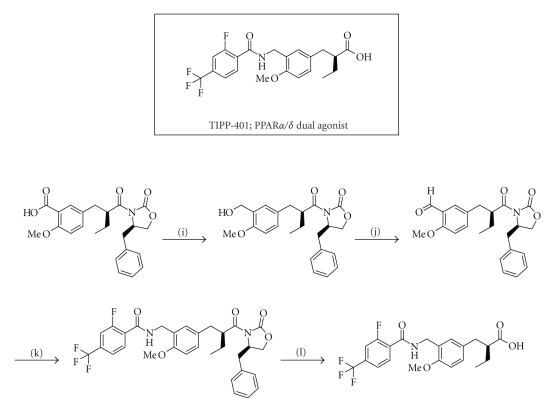
Chemical structure of our PPAR*α*/*δ*-dual agonist TIPP-401, and the synthetic route. Reagents and conditions: (i) BH_3_-tetrahydrofuran, THF, 0°C, (j) activated MnO_2_, CH_2_Cl_2_, r.t., (k) 2-fluoro-4-trifluoromethylbenzamide, triethylsilane, trifluoroacetic acid, toluene, reflux, (l) LiOH H_2_O, H_2_O_2_, THF-H_2_O, 0°C.

We selected 33 as the next lead compound, and performed further chemical modifications, focusing especially on the hydrophobic tail part of the molecule, and obtained a PPAR*α*/*δ*-dual agonist termed TIPP-401 ((*S*)-2-{3-[(2-fluoro-4-trifluoromethylbenzoylamino)methyl]-4-methoxybenzyl}butyric acid). The synthetic route is summarized in Scheme 2. We found that the introduction of a fluorine atom affected the PPAR transactivation activity or selectivity (Table 4). Compounds 38 and 39, which have a fluorine atom at the *ortho*- or *meta*-position of benzene in the hydrophobic tail part, respectively, exhibited more potent PPAR*α* and PPAR*δ* transactivation activities than those of the nonfluorinated compound. The position of the distal benzene substituents is crucial, since compound 40, which has a fluorine atom at the *para*-position and a trifluoromethyl group at the *meta*-position, showed considerably decreased PPAR transactivation activity. This is consistent with the previously obtained SAR result that steric bulkiness at the *para*-position is an important factor for potent PPAR*α* transactivation activity.

Considering these results, we prepared optically active derivatives, 41 (TIPP-401), 42, and 43. As can be seen from Table 4, a clear enantio-dependency of the transactivation activity towards the PPAR*α* and PPAR*δ* isoforms was found. Compound 42, which has (*S*) configuration, exhibited potent transactivation activity towards both PPAR*α* and PPAR*δ*, while the antipodal (*R*) isomer 43 exhibited far less potency. Therefore, we concluded that both PPAR*α* and PPAR*δ* transactivation activities reside almost exclusively in the (*S*)-enantiomer, and both TIPP-401 and 42 show dual-agonist activity toward PPAR*α* and PPAR*δ*. The synthetic route to TIPP-401 is shown in Scheme 2.

**Figure 4:**
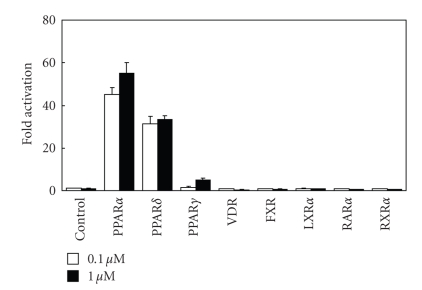
Cross-nuclear receptor reactivity of 0.1 *μ*M, and 1 *μ*M TIPP-401.

In order to investigate the nuclear receptor selectivity (cross-reactivity) of the representative compound TIPP-401, we determined the transactivation activity of TIPP-401 on representative nuclear receptors (PPARs, VDR, FXR, LXR*α*, RAR*α*, and RXR*α*). As can be seen from Figure 4, TIPP-401 seems to be specific for PPAR*α* and PPAR*δ* because it did not significantly activate VDR, PPAR*γ*, LXR*α*, RAR*α*, or RXR*α* at concentrations up to 1 *μ*M under the experimental conditions used. These results indicate that although the ligand binding domains of nuclear receptors are similar, there are distinct structural requirements for preferential binding to both PPAR*α* and PPAR*δ*.

## 8. PPAR*δ*-SELECTIVE AGONIST: TIPP-204

Our next target was a PPAR*δ*-selective agonist. As described above, the availability of PPAR$\overset{˜}{\delta }$ knockout animals and selective ligands, especially GW-50151 (3), developed by GlaxoSmithKline, prompted to examine the involvement of PPAR*δ* in fatty acid metabolism, insulin resistance, reverse cholesterol transport, inflammation, and so on. For example, ligand-mediated PPAR*δ* activation significantly increased HDL cholesterol levels, possibly in association with decreased lipoprotein lipase activity, in insulin-resistant middle-aged obese rhesus monkeys [38].** In a primate model of the metabolic syndrome, PPAR*δ* activation lowered plasma insulin levels, without any adverse effect on glycemic control [38]. Similarly, in the case of ob/ob mice, PPAR*δ* activation markedly improved glucose tolerance and insulin resistance [38]. All these observations suggest that PPAR*δ* may be an effective target for the treatment of metabolic syndrome.

**Figure 5:**
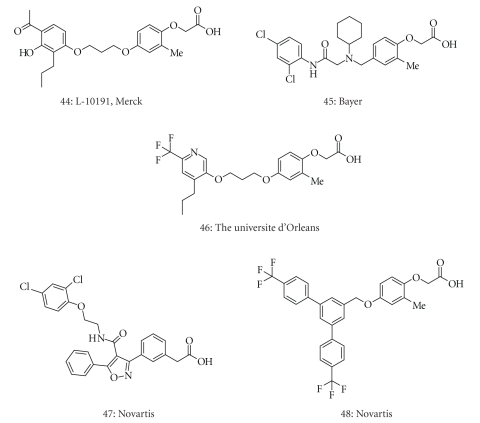
Structures of representative, recently reported PPAR*δ*-selectiv agonists.

Several PPAR$\overset{˜}{\delta }$ selective agonists (44–48) have been reported in the literature (Figure 5) after the disclosure of GW-501516 (3), though most are derivatives of GW-501516 (3) and L-165041 (44) (Merck's compound), that is, (2-methyl)phenoxyacetic acid derivatives [39–44]. As a part of our continuing research directed toward the structural development of characteristic subtype-selective PPAR agonists, we planned to construct phenylpropanoic acid-type PPAR$\overset{˜}{\delta }$ selective agonists, based on the PPAR*α*/*δ* dual agonist, TIPP-401 as a lead compound.

To create PPAR*δ*-selective agonists, we took into account the results of X-ray crystallographic analyses of PPAR*δ* complexed with a natural unsaturated fatty acid, eicosapentaenoic acid (EPA) [45]. The PPAR ligand-binding pocket forms a large Y-shaped cavity which extends from the C-terminal helix to the *β*-sheet lying between helix 3 and helix 6. EPA binds to the cavity in two distinct conformations, that is, *tail-up* and *tail-down* conformations. The carboxyl group and the first eight carbon units take almost the same configuration in both conformations. However, the distal hydrophobic tail part of the tail-up conformer of EPA was bent upwards into the upper cavity of the Y-shaped pocket, while in the tail-down conformer, the hydrophobic tail part was bent downwards into the bottom cavity of the Y-shaped pocket. Contrary to the case of PPAR*δ*, none of the PPAR*α* and PPAR*γ* agonists whose binding structures have been solved by X-ray crystallography takes the tail-up conformation, although the reason for this is not known.** However, we speculated that the aminoacid(s) forming the entrance to the upper cavity might be bulkier in PPAR*α* and PPAR*γ* than in PPAR*δ*. We previously suggested that our PPAR*α*-selective agonist KCL might take a tail-down conformation, based on molecular modeling studies of the KCL-PPAR*α* complex and the results of site-directed mutagenesis studies of PPAR*α* with our PPAR agonists. The PPAR*α*/*δ* dual agonist TIPP-401 was also considered to dock into the downward cavity of PPAR*α*, because Ile272, which is located on the lower half of helix 3, is also critical for potent PPAR*α* transactivation by TIPP-401.

**Scheme 3:**
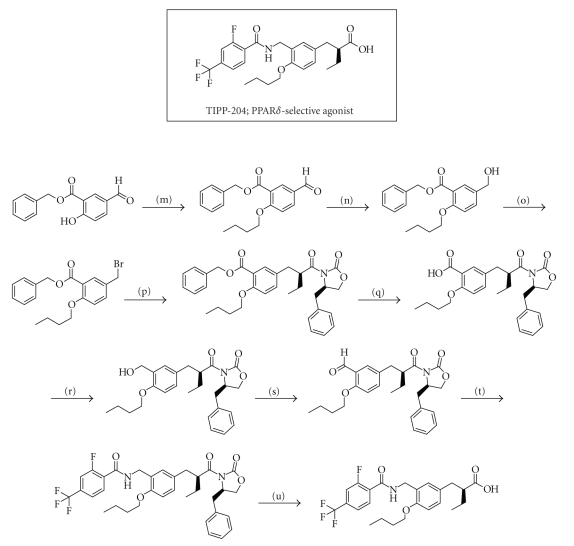
The synthetic route to TIPP-204. Reagents and conditions: (m) nBuI, K_2_CO_3_, DMF, rt., (n) NaBH_4_, EtOH, rt., (o) PBr_3_, ether, 0°C, (p) (1) (*R*)-4-benzyl-3-butyryloxazolidin-2-one, LiHMDS, THF, −40°C−−10°C, (2) benzyl 5-bromomethyl-2-butoxybenzoate, THF, −40°C−−10°C, (q) H_2_, 10% Pd–c, AcOEt, rt., (r) BH_3_-tetrahydrofuran, THF, 0°C, (s) activated MnO_2_, CH_2_Cl_2_, rt., (t) 2-fluoro-4-trifluoromethylbenzamide, triethylsilane, trifluoroacetic acid, toluene, reflux, (u) LiOH H_2_O, H_2_O_2_, THF-H_2_O, 0° C.

Based on these insights, we hypothesized that if we could connect one more sterically bulky hydrophobic side chain to the backbone of TIPP-401, directed towards the upper cavity of PPAR*δ*, it should have the effect of strengthening the PPAR*δ* activity, while weakening the PPAR*α* activity.** Based on our previously reported binding model of KCL, a methoxy group at the 4-position was expected to be directed towards the upper cavity, so we prepared various 3-(4-alkoxyphenyl)propanoic acids, and found the compound termed TIPP-204 ((*S*)-2-{3-[(2-fluoro-4-trifluoromethylbenzoylamino)methyl]-4-buthoxy- benzyl}butyric acid). The structure and the method of preparation are summarized in Scheme 3. We obtained clear SAR confirming that the subtype-selectivity largely depends on the nature of the substituents, as expected.

That is, as regards PPAR*α*, introduction of a short-chain alkoxy group was found to be favorable for the transactivation activity, that is, the ethoxy (50) and methoxy (49) derivatives exhibited the most potent activity. In contrast, a longer alkoxy group was preferable in the case of PPAR*δ* transactivation activity, and the *n*-butoxy (52) and *n*-propoxy (51) derivatives were the most potent (Table 5). These results are consistent with the working hypothesis that the shape and the environment of the hydrophobic cavity hosting the alkoxy group at the *para*-position differ somewhat among these PPAR subtypes. These compounds were basically weak agonists for the PPAR*γ* subtype, because each compound exhibited an EC_50_ value of micromolar order or less.

As described above, the introduction of a fluorine atom on the distal benzene ring, especially at the 2-position, was found to enhance the PPAR transactivation activity, as in TIPP-401. Therefore, we prepared fluorinated compounds 55–57. As expected, these compounds exhibited enhanced PPAR*α* and PPAR*δ* transactivation activities as compared with the nonfluorinated compounds. We found that the PPAR*δ* transactivation activity of 57 is comparable with that of GW-501516 in our assay system, and the selectivity indexes for PPAR*δ* over both PPAR*α* and PPAR*γ* are more than 100-fold (Table 5).

As mentioned above, the substituent at the *α*-position of the carboxyl group is also important for the potency in the case of PPAR*α*, and therefore we investigated the effect of substitution at this position in the present series. As regards PPAR*α*, introduction of an ethyl group (49) or a methyl group (59) was favorable for the transactivation activity, and further elongation of the substituent decreased the activity. Similarly, an ethyl group (49) or an *n*-propyl group (60) was favorable for PPAR*δ* transactivation activity, and further elongation of the substituent decreased the activity (Table 5). These results may mean that the shape and the environment of the cavity hosting the alkyl group located at the *α*-position of the carboxyl group are similar in PPAR*α* and PPAR*δ*.

**Figure 6:**
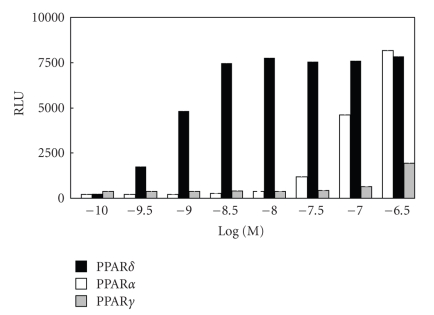
Dose-dependency of TIPP-204 for transactivation of PPARs.

Considering these results, we then prepared the optically active derivatives 65 (*S*-isomer)(TIPP-204) and 66 (*R*-isomer). As expected, clear enantio-dependency of the transactivation activity towards the PPAR subtypes was found, and TIPP-204, which has (*S*)-configuration, exhibited more potent transactivation activity than the antipodal (*R*)-isomer, 66 (Table 5). Therefore, we concluded that the activity also resides primarily in the (*S*)-enantiomer, but the degree of the enantio-selectivity is less apparent than in the case of the PPAR*α*/*δ* dual agonist, TIPP-401. TIPP-204 exhibited extremely potent PPAR*δ* transactivation activity, comparable with or even superior to that of the known PPAR*δ*-selective agonist GW-501516, and its PPAR subtype selectivity was also high (Figure 6).

**Figure 7:**
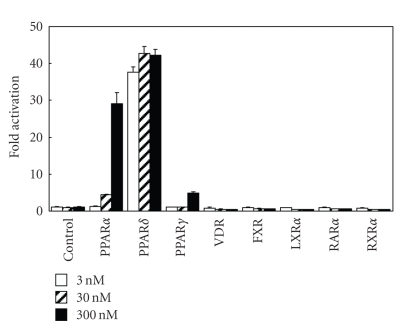
Nuclear receptor selectivity (cross reactivity) of TIPP-204.

To investigate the nuclear receptor selectivity (cross-reactivity) of TIPP-204, we analyzed the transactivation activity of TIPP-204 on representative nuclear receptors (PPARs, VDR, FXR, LXR*α*, RAR*α*, and RXR*α*) in the same way as with TIPP-401 (Figure 7). TIPP-204 seems to be specific for PPAR*δ* (and to a lesser extent PPAR*α*) because it did not significantly activate VDR, PPAR*γ*, LXR*α*, RAR*α*, or RXR*α* at concentrations up to 300 nM (more than 300-fold higher concentration as compared to the EC_50_ of TIPP-204) under the experimental conditions used. These results indicate that, although the ligand-binding domains of nuclear receptors are similar, there are distinct structural requirements for preferential binding of TIPP-204 to PPAR*δ*.

**Figure 8:**
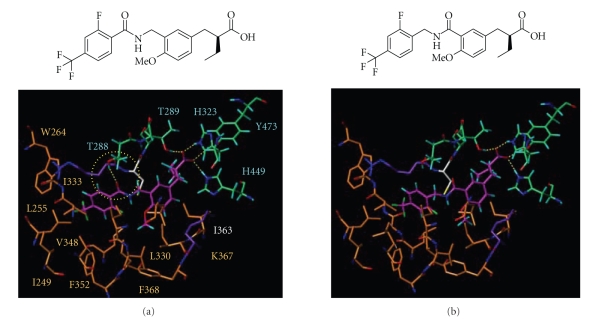
Predicted mode of binding the amide derivative and the reversed-amide derivative to PPAR*δ*. Orange: hydrophobic aminoacids. Green: hydrophilic aminoacids. Magenta: amide derivative and reversed-amide derivative. Hydrogen bonds are shown as yellow dotted lines.

**Figure 9:**
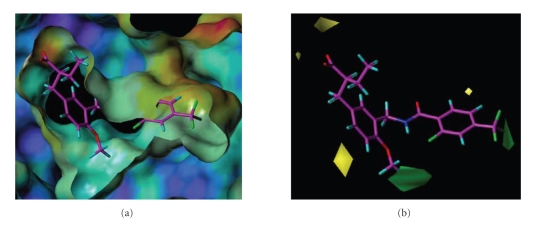
Comparison of the CoMFA counterplot of the steric field based on TIPP-401 (right) and the superimposition of TIPP-401 on the ligand-binding domain of PPAR*δ* (left). The CoMFA steric counter map is shown in green and yellow. Green: areas in which bulky atomic groups are sterically favorable for the activity. Yellow: areas in which bulky atomic groups are unfavorable for the activity.

**Figure 10:**
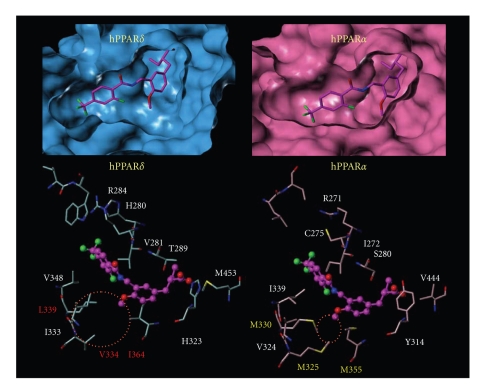
Predicted mode of binding of TIPP-401 to PPAR*δ* and PPAR*α*. Upper figures represent van der Waals surface contact of ligand-binding cavity, and the lower figure represent detailed component aminoacids.

We have successfully obtained a potent and selective, structurally novel PPAR*δ* agonist, TIPP-204. In order to investigate the structure-activity relationship, and the reason for the PPAR*δ* selectivity, we analyzed the three-dimensional structure-activity relationship by means of the comparative molecular field analysis (CoMFA) method, and a molecular modeling study (Figures 8–10). (Comparative molecular field analysis (CoMFA) was used to construct a three-dimensional quantitative structure-activity relationships model. The atomic charges of each conformer were calculated using the semiempirical method MNDO with electrostatic potential-derived point charges (MNDO/ESP) in MOPAC2002. Conventional CoMFA was performed using the QSAR option implemented in the SYBYL package. CoMFA fields were derived in a 3D cubic lattice with a grid spacing of 2 Å and extending 4 Å beyond the aligned molecules in all directions. CoMFA steric (Lennard-Jones 6-12 potential) field energies and CoMFA electrostatic (Coulombic potential) fields were calculated using a probe atom with the van der Waals properties of sp^3^ carbon and a charge of +1.0. CoMFA electrostatic fields were calculated with a distance-dependent dielectric at each lattice point. The SYBYL energy cutoff of 30 kcal/mol was used. In CoMFA calculation, potential functions (a Lennard-Jones potential and a Coulombic potential) are very steep near the van der Waals surface, causing rapid change, so that the use of cut-off values is required. The poses of ligands generated from the Glide program were used to carry out partial-least-square (PLS) regression analyses. The CoMFA fields were used as independent variables and the logarithm of the reciprocal of EC_50_ was used as the dependent variable in PLS regression analyses. The optimal number of components in the PLS model was determined using the cross-validated coefficient *r* ^2^ (called *q* ^2^) values obtained from the leave-one-out cross-validation technique. The PLS model with the highest *q* ^2^ values was then selected to derive 3D QSAR models and the poses of ligands with respect to the PPAR*δ* LBD.) Comparison of the CoMFA counterplots with the crystal structure of the PPAR*δ* ligand-binding domain provided information about how structural changes of the agonists affect their activities. As can be seen in Figure 8, hydrogen-bonding interaction was observed between carbonyl oxygen of the reversed-amide type linker and threonine 288 (T288) of PPAR*δ* (Figure 8 left panel), while such a hydrogen-bonding interaction was not found between carbonyl oxygen of the amide-type linker and T288 (Figure 8 right panel). This might be one of the reasons why the change of the linker from amide type to reversed-amide type enhanced the PPAR*δ* transactivation activity by 10-fold.

In this CoMFA model (Figure 9), the introduction of a sterically bulky group near the methoxy group at the 4-position in the present series favors the activity, and this was deduced to be related to the presence of the upper cavity in the Y-shaped ligand-binding domain of PPAR*δ*. Based on our molecular modeling (Figure 10), we speculated that the side-chain butoxy group of TIPP-204 fits into the upper cavity of hPPAR*δ* formed by the hydrophobic aminoacids V334, L339, and I364. In the case of hPPAR*α*, the corresponding cavity is composed of sterically bulkier aminoacids, M325, M330, and M355. Indeed, the volume of the hPPAR*α* upper cavity was calculated to be only one third of that of the hPPAR*δ* upper cavity, and we considered that it might not readily accommodate the bulky butoxy group of TIPP-204. In order to confirm this idea, an X-ray crystallographic analysis in combination with molecular modeling is in progress.

## 9. CONCLUDING REMARKS AND FUTURE DIRECTIONS

In this minireview, we have described our PPAR ligands based on 3,4-disubstituted phenylpropanoic acid structure as a versatile template for subtype-selective PPARs ligands, based on our ligand superfamily working hypothesis. We also describe their pharmacological evaluation.** We succeeded in obtaining three kinds of subtype-selective PPARs ligands, that is, the PPAR*α* selective agonist KCL, the PPAR*α*/*δ*-dual agonist TIPP-401, and the PPAR*δ* selective agonist TIPP-204. The structure-activity relationships among ligands for PPAR*α* and PPAR*δ* were well charcterized, and are summarized in Figure 10.

Basically, our series of compounds showed weak activity against the PPAR*γ* subtype. However, considering the moderately high-sequence similarity among PPARs, it should be possible to obtain greater activity. So, we are conducting further chemical modification studies directed towards PPAR*γ* activity. Some novel structural requirements for PPAR*γ* activity have already been identified. We expect to report 3,4-disubstituted phenylpropanoic acid-type PPAR-pan agonists in the near future.

## Figures and Tables

**Figure 11:**
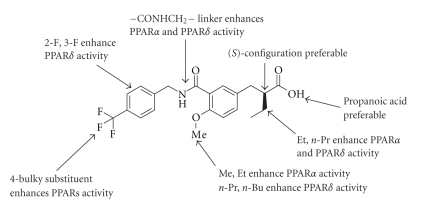
SAR summary of the present series of compounds.

**Table 1:** SAR 1: effect of the acidic partial structure in the present series of compounds.

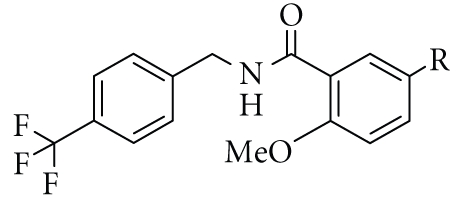				
		Transactivation activity		
		EC_50_ (nM)^(a)^		
No.	R	PPAR*α*	PPAR*δ*	PPAR*γ*
7	COOH	ia^(b)^	ia	ia
8	CH_2_COOH	ia	ia	ia
9	(CH_2_)_2_COOH	1300	ia	ia
10	CH_2_CH(Me)COOH^(c)^	240	2800	ia
11	CH_2_CH(Et)COOH^(c)^	40	3600	1000
12	CH_2_CH(*n*-Pr)COOH^(c)^	360	2400	ia
13	CH_2_CH(*i*-Pr)COOH^(c)^	290	ia	ia
14	CH_2_CH(*n*-Bu)COOH^(c)^	1000	ia	2500
15	CH_2_CH(Ph)COOH^(c)^	ia	ia	ia
16	CH_2_CH(OMe)COOH^(c)^	230	ia	ia
17	CH_2_CH(OEt)COOH^(c)^	1600	3000	2800
18	CH_2_CH(OPh)COOH^(c)^	ia	ia	ia
19	CH_2_C(Me)_2_COOH	2900	ia	ia
20	CH_2_C(Et)_2_COOH	2800	ia	ia
21	CH_2_CH(SEt)COOH^(c)^	1600	3000	2800
22	CH_2_CH(SPh)COOH^(c)^	ia	ia	ia
23	CH_2_CH(SBn)COOH^(c)^	ia	ia	ia

	KRP-297	1000	ia	800

^(a)^ Compounds were screened for agonist activity on PPAR-GAL4 chimeric receptors in transiently transfected CHO-K1 cells as described.** EC_50_ value is the molar concentration of the test compound that causes 50% of the maximal reporter activity, ^(b)^ “ia” means inactive at the** concentration of 10 *μ*M, ^(c)^ assayed as a racemate.

**Table 2:** SAR 2: effect of the linker partial structure in the present series of compounds.

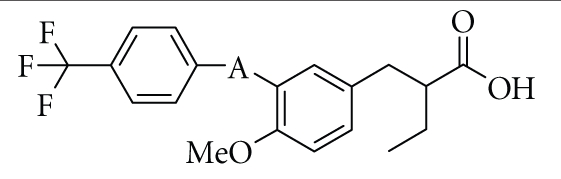				
		Transactivation activity		
		EC_50_ (nM)^(a)^		
No.	A	PPAR*α*	PPAR*δ*	PPAR*γ*
11	CH_2_NHCO^(c)^	40	3600	1000
24	NHCO^(c)^	6000	ia^(b)^	ia
25	(CH_2_)_2_NHCO^(c)^	740	1500	ia
26	CH_2_N(Me)CO^(c)^	ia	ia	ia
27	CH_2_CONH^(c)^	130	ia	ia
28	NHCONH^(c)^	ia	ia	ia
29	NHCOCH_2_^(c)^	20	ia	ia
30	CH_2_CH_2_CO^(c)^	320	ia	7400
31	CH_2_CH_2_CH_2_^(c)^	860	640	ia
32	CH_2_CH_2_O^(c)^	680	400	ia
33	CONHCH_2_^(c)^	40	120	10000
34	CH_2_NHCH_2_^(c)^	4900	8000	ia

^(a), (b), (c)^ See footnotes of Table 1.

**Table 3:** Species differences in the transactivation of PPAR*α* isoforms.

	Transactivation activity		
	EC_50_ (nM)^(a)^		
Compd.	Human	Dog	Rat
KCL	60	160	5200
fenofibrate	41000	50000	49000

^(a)^ See footnote of Table 1.

**Table 4:** In vitro functional PPAR transactivation activity of substituted phenylpropanoic acids.

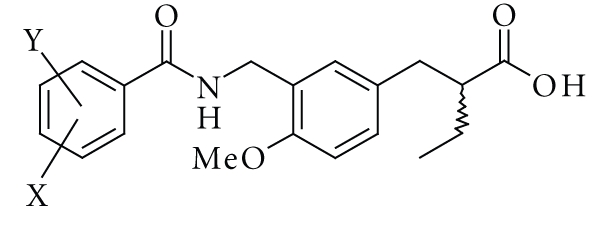						
				Transactivation activity		
				EC_50_ (nM)^(a)^		
No.	X	Y	stereo	PPAR*α*	PPAR*δ*	PPAR*γ*
33	4-CF_3_	H	rac	19	200	2600
38	4-CF_3_	2-F	rac	10	24	2200
39	4-CF_3_	3-F	rac	11	51	6000
40	3-F	4-CF_3_	rac	250	2000	ia^(b)^
41	4-CF_3_	2-F	*S*	10	12	1900
42	4-CF_3_	3-F	*S*	12	23	4900
43	4-CF_3_	3-F	*R*	150	840	ia^(b)^

^(a)^ Compounds were screened for agonist activity on PPAR-GAL4 chimeric receptors in transiently transfected HEK-293 cells. The EC_50_ value is the molar** concentration of the test compound that causes 50% of the maximal reporter activity, ^(b)^ “ia” means inactive at the concentration of 10 *μ*M.

**Table 5:** PPARs transactivation activity of the present series of compounds.

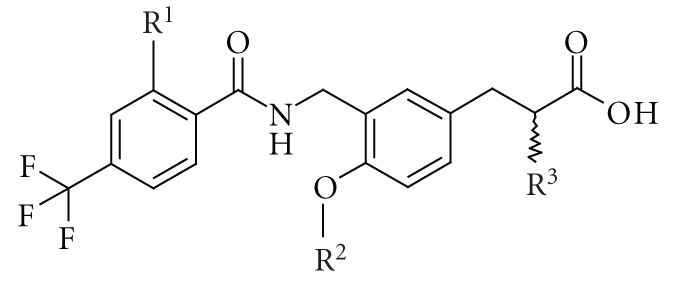							
					Transactivation activity		
					EC_50_ (nM)^(a)^		
No.	R^1^	R^2^	R^3^	stereo	PPAR*α*	PPAR*δ*	PPAR*γ*
49	H	Me	Et	rac	18	170	2600
50	H	Et	Et	rac	12	15	1800
51	H	*n*-Pr	Et	rac	77	6.8	1300
52	H	*n*-Bu	Et	rac	520	4.8	1300
53	H	*n*-Hexyl	Et	rac	1200	45	2300
54	H	Bn	Et	rac	>10000	110	6200
55	F	Me	Et	rac	8.2	21	2200
56	F	*n*-Pr	Et	rac	41	1.7	650
57	F	*n*-BU	Et	rac	280	1.9	1400
58	H	Me	H	rac	210	3000	>10000
59	H	Me	Me	rac	19	210	>10000
60	H	Me	*n* Pr	rac	70	120	3600
61	H	Me	*n* BU	rac	230	700	2400
62	F	Me	*n* Pr	rac	29	40	1500
63	F	Me	*n* BU	rac	140	220	1000
64	F	*n*-BU	*n* Pr	rac	820	5.1	1000
65	F	*n*-BU	Et	*S*	250	0.91	1100
66	F	*n*-BU	Et	*R*	620	8.2	7600

GW-501516					1000	1.8	8600

^(a)^ Compounds were screened for agonist activity on PPAR-GAL4 chimeric receptors in transiently transfected HEK-293 cells. The EC_50_ value is the molar concentration of the test compound that causes 50% of the maximal reporter activity.
